# Molecular characterization and clonal dynamics of nosocomial *bla*_OXA-23_ producing XDR *Acinetobacter baumannii*

**DOI:** 10.1371/journal.pone.0198643

**Published:** 2018-06-11

**Authors:** Sabrina Royer, Paola Amaral de Campos, Bruna Fuga Araújo, Melina Lorraine Ferreira, Iara Rossi Gonçalves, Deivid William da Fonseca Batistão, Rebecca Tavares e Silva Brígido, Louise Teixeira Cerdeira, Luiz Gustavo Machado, Cristiane Silveira de Brito, Paulo Pinto Gontijo-Filho, Rosineide Marques Ribas

**Affiliations:** 1 Institute of Biomedical Sciences (ICBIM), Laboratory of Molecular Microbiology, Federal University of Uberlandia, Uberlandia, Minas Gerais, Brazil; 2 School of Medicine (FAMED), Federal University of Uberlandia, Uberlandia, Minas Gerais, Brazil; 3 National Reference Center for Sanitary Dermatology and Leprosy (CREDESH) Clinical Hospital Federal University of Uberlândia, Uberlândia, Minas Gerais, Brazil; 4 Department of Clinical Analysis, School of Pharmacy, University of São Paulo, São Paulo, Brazil; Tianjin University, CHINA

## Abstract

The emergence of infections associated to new antimicrobial resistance in *Acinetobacter baumannii* (Ab) genotypes represents a major challenge. In this context, this study aimed to determine the diversity of resistance mechanisms and investigate clonal dissemination and predominant sequence types (STs) in multidrug-resistant Ab strains of clinical (tracheal aspirate, n = 17) and environmental (surface, n = 6) origins. Additionally, the major clones found in clinical (A) and environmental (H) strains had their complete genomes sequenced. All strains were submitted to polymerase chain reactions (PCR) for the detection of the IS*Aba1*/*bla*_OXA-51-like_ and IS*Aba1*/*bla*_OXA-23-like_ genes, while the expression of genes encoding the *carO* porin, AdeABC (*adeB*), AdeFGH (*adeG*), and AdeIJK (*adeJ*) efflux pumps was determined by real time PCR (qPCR). Most of the strains were characterized as extensively drug-resistant (XDR) with high minimal inhibitory concentrations (MICs) detected for tigecycline and carbapenems. Associations between IS*Aba1*/OXA-51 and IS*Aba1*/OXA-23 were observed in 91.3% and 52.2% of the strains, respectively. Only the *adeB* gene was considered hyper-expressed. Furthermore, most of the strains analyzed by the MuLtilocus Sequence-Typing (MLST) were found to belong to the clonal complex 113 (CC113). In addition, a new ST, ST1399, belonging to CC229, was also discovered herein. Strains analyzed by whole genome sequencing presented resistance genes linked to multidrug-resistance phenotypes and confirmed the presence of Tn*2008*, which provides high levels carbapenem-resistance.

## Introduction

*Acinetobacter baumannii* (Ab) is one of the most successful pathogens responsible for hospital-acquired infections worldwide, mainly in immunocompromised patients and those admitted to intensive care units (ICUs) [1,2].

Carbapenem resistance in Ab is most frequently mediated by intrinsic (OXA-51-like) or acquired (mainly OXA-23-like) oxacillinases [3]. The hyper-expression of oxacillinases is often associated to insertion sequences (ISs), such as the IS*Aba1*sequence, located upstream of the OXA genes [4–6]. In addition, the increased expression of chromosomal genes of resistance-nodulation-cell division (RND)-type efflux systems [2,7] and the decreased expression of certain outer membrane channel-forming proteins [8,9] play an important role in Ab multidrug resistance. The relative contribution of these resistance mechanisms remains poorly assessed in clinical contexts [8].

Regarding the clonal nature and the spread of different sequence types (STs), both Brazilian and Latin American studies conducted between 2011 and 2016 demonstrated that the most widespread Ab strains belong to CC113 and CC109 [10–16], characterized by MuLtilocus Sequence-Typing—University of Oxford (MLST-UO). Other studies worldwide indicate that multidrug-resistant Ab is related to CC92 [17,12,18–19]. Tracking the evolution and clonal dissemination of carbapenem resistance in Ab isolates is important to support the implementation of control strategies, which is only possible through a comprehensive understanding of the complete genome of these strains.

In this context, the aim of the present study was to investigate the clonal dissemination and genetic basis of resistance among multidrug-resistant Ab strains recovered from an adult ICU in Brazil. Additionally, one clinical and one environmental strain belonging to the prevalent clones had their complete genomes sequenced.

## Material and methods

### Bacterial strains and setting

The origin and epidemiological characteristics of the strains used herein are described in Table 1. Clinical and environmental *A*. *baumannii* isolates were obtained from patients with ventilator-associated pneumonia (VAP) and the environments around their beds (bedside table, bed rail and door knob), respectively. The strains were obtained from April 2011 to June 2012, in a 30-bed clinical-surgical intensive care unit (ICU) at the Clinical Hospital belonging to the Federal University of Uberlandia, Minas Gerais, Brazil. The 23 isolates investigated herein were selected according to their resistance profile and pulsotype, obtained through Pulsed Field Gel Electrophoresis (PFGE), as published previously [20].

**Table 1:** Molecular characterization by polymerase chain reaction (PCR) of resistance determinants and distribution of MICs to carbapenems and tigecycline in 23 strains of *A*. *baumannii* recovered from endotracheal aspirate and environment in an adult intensive care unit.

Strains^1^/Source	Resistance genes	Porines genes	Efflux pumps genes	MDR^2^/XDR^3^	MIC^4^ (μg/m) IPM^5^/MEM^6^	MIC (μg/mL) TGC^7^	MIC^8^ (μg/mL) TGC	PFGE^9^ profile
**13/EA**^10^	*bla*_OXA-51_; *bla*_OXA-23;_ *ISAba1; ISAba1/OXA-23; ISAba1/OXA-51*	*carO*; *omp33-36*	*adeB*; *adeG*; *adeJ*	XDR	>32/>32	>256	64	A
**15/EN**^11^	*bla*_OXA-51_; *bla*_OXA-23;_ *ISAba1; ISAba1/OXA-23; ISAba1/OXA-51*	*carO*; *omp33-36*	*adeB*; *adeG*; *adeJ*	XDR	>32/>32	96	64	H
**17/EN**	*bla*_OXA-51_; *bla*_OXA-23;_ *ISAba1; ISAba1/OXA-23; ISAba1/OXA-51*	*carO*; *omp33-36*	*adeB*; *adeG*; *adeJ*	XDR	>32/>32	48	64	H
**20/EN**	*bla*_OXA-51_; *bla*_OXA-23;_ *ISAba1; ISAba1/OXA-23; ISAba1/OXA-51*	*carO*; *omp33-36*	*adeB*; *adeG*; *adeJ*	MDR	>32/>32	24	64	E
**2/EA**	*bla*_OXA-51_; *bla*_OXA-23;_ *ISAba1; ISAba1/OXA-23; ISAba1/OXA-51*	*carO*; *omp33-36*	*adeB*; *adeG*; *adeJ*	XDR	24/>32	>256	64	A
**16/EN**	*bla*_OXA-51_; *bla*_OXA-23;_ *ISAba1; ISAba1/OXA-23; ISAba1/OXA-51*	*carO*; *omp33-36*	*adeB*; *adeG*; *adeJ*	XDR	24/>32	96	64	H
**18/EN**	*bla*_OXA-51_; *bla*_OXA-23;_ *ISAba1; ISAba1/OXA-23; ISAba1/OXA-51*	*carO*; *omp33-36*	*adeB*; *adeG*; *adeJ*	XDR	16/>32	64	64	G
**19/EN**	*bla*_OXA-51_; *bla*_OXA-23;_ *ISAba1; ISAba1/OXA-23; ISAba1/OXA-51*	*carO*; *omp33-36*	*adeB*; *adeG*; *adeJ*	XDR	12/32	48	64	H
**1/EA**	*bla*_OXA-51_; *bla*_OXA-23;_ *ISAba1; ISAba1/OXA-23; ISAba1/OXA-51*	*carO*; *omp33-36*	*adeB*; *adeG*; *adeJ*	XDR	12/16	>256	64	A
**9/EA**	*bla*_OXA-51_; *bla*_OXA-23;_ *ISAba1; ISAba1/OXA-23; ISAba1/OXA-51*	*carO*; *omp33-36*	*adeB*; *adeG*; *adeJ*	MDR	4/3	8	64	C
**5/EA**	*bla*_OXA-51_; *bla*_OXA-23;_ *ISAba1/OXA-23; ISAba1/OXA-51*	*carO*; *omp33-36*	*adeB*; *adeG*; *adeJ*	XDR	8/12	48	64	D
**3/EA**	*bla*_OXA-51_; *bla*_OXA-23;_ *ISAba1; ISAba1/OXA-51*	*carO*; *omp33-36*	*adeB*; *adeG*; *adeJ*	XDR	>32/>32	>256	64	A
**12/EA**	*bla*_OXA-51_; *bla*_OXA-23;_ *ISAba1; ISAba1/OXA-51*	*omp33-36*	*adeB*; *adeG*; *adeJ*	XDR	>32/>32	24	64	C
**8/EA**	*bla*_OXA-51_; *bla*_OXA-23;_ *ISAba1; ISAba1/OXA-51*	*carO*; *omp33-36*	*adeB*; *adeG*; *adeJ*	XDR	24/32	64	64	A
**6/EA**	*bla*_OXA-51_; *bla*_OXA-23;_ *ISAba1; ISAba1/OXA-51*	*carO*; *omp33-36*	*adeB*; *adeG*; *adeJ*	XDR	16/24	256	64	A
**11/EA**	*bla*_OXA-51_; *bla*_OXA-23;_ *ISAba1; ISAba1/OXA-51*	*carO*; *omp33-36*	*adeB*; *adeG*; *adeJ*	XDR	8/6	>256	64	A
**14/EA**	*bla*_OXA-51_; *bla*_OXA-23_; *ISAba1/OXA-23*	*carO*; *omp33-36*	*adeB*; *adeG*; *adeJ*	XDR	>32/>32	256	64	G
**4/EA**	*bla*_OXA-51_; *bla*_OXA-23_*; ISAba1/OXA-51*	*carO*; *omp33-36*	*adeB*; *adeG*; *adeJ*	XDR	>32/>32	>256	64	A
**10/EA**	*bla*_OXA-51_; *bla*_OXA-23_*; ISAba1/OXA-51*	*omp33-36*	*adeB*; *adeG*; *adeJ*	XDR	>32/>32	32	32	B
**7/EA**	*bla*_OXA-51_; *bla*_OXA-23_*; ISAba1/OXA-51*	*omp33-36*	*adeB*; *adeG*; *adeJ*	XDR	16/>32	24	32	A
**23/EA**	*bla*_OXA-51_; *ISAba1; ISAba1/OXA-51*	*omp33-36*	*adeB*; *adeG*; *adeJ*	MDR	1/3	128	64	G
**21/EA**	*bla*_OXA-51_; *ISAba1; ISAba1/OXA-51*	*carO*; *omp33-36*	*adeB*; *adeG*; *adeJ*	No MDR^12^	0,75/3	6	64	G
**22/EA**	*bla*_OXA-51_; *ISAba1*	*carO*; *omp33-36*	*adeB*; *adeG*; *adeJ*	No MDR	3/6	8	64	F

^1^All negative strains for *bla*_OXA-24_, *bla*_OXA-58_ and *bla*_OXA-143_ genes;

^2^MDR, Multidrug-resistant;

^3^XDR, Extensively drug-resistant;

^4^MIC, Minimum Inhibitory Concentration—Etest^®^;

^5^IPM, Imipenem (0,002–32μg/mL);

^6^MEM, Meropenem (0,002–32μg/mL);

^7^TGC, Tigecycline (0,016–256μg/mL);

^8^MIC, Minimum Inhibitory Concentration—Broth microdilution (0,125–256μg/mL);

^9^PFGE, Pulsed Field Gel Electrophoresis;

^10^EA, Endotracheal aspirate;

^11^EN, Environment.

^12^No MDR, strains do not present resistance to three or more antimicrobial categories.

### Clinical microbiology

Resistance to tigecycline, imipenem and meropenem was determined by the Etest^®^ method according to the manufacturer’s guidelines (AB Biodisk, Solna, Sweden). To confirm the results of Etest^®^ for tigecycline, the broth microdilution technique was performed. All the resistance tests and the quality control protocols were done in accordance with the Clinical and Laboratory Standards Institute recommended practices [21]. Since there were no breakpoints available for tigecycline for *Acinetobacter* spp., US Food and Drug Administration (FDA) tigecycline breakpoints listed for Enterobacteriaceae (≤ 2, 4 and ≥ 8 μg/ml for susceptible, intermediate and resistant strains, respectively) were applied. Multidrug-resistant (MDR) was defined as resistance to three or more categories of antibiotics while extensively drug-resistant (XDR) was defined as non-susceptibility to at least one agent in all but two or fewer antimicrobial categories, according to Magiorakos and collaborators [22].

### Polymerase chain reaction

The DNA extraction was performed by thermal lysis and a conventional PCR assay was performed for 23 isolates to detect the following genes: IS*Aba1*/*bla*_OXA-51-like_, IS*Aba1*/*bla*_OXA-23-like_, *omp33*-*36*, *carO* (29 kDa), *adeB*, *adeG* and *adeJ*, using previous published primers (S1 Table) [23,4,24,25,26,2]. The amplified PCR products were visualized by electrophoresis on 1.5% agarose gels using the photo documentation System L-Pix EX (Loccus Biotechnology, Brazil).

### Relative quantification of CarO porin and *adeB*, *adeG* and *adeJ* efflux system genes by real time PCR (qPCR)

Out of a total of 23 strains with their clonals profiles previously evaluated [20] 10 were selected for analysis by qPCR. The *carO*, *adeB*, *adeG* and *adeJ* transcription was evaluated by qPCR using Power-SYBR Green PCR Master Mix (Applied Biosystems). The single-copy housekeeping *rpoB* gene from Ab was used as endogenous gene for normalization (S1 Table) [2,27]. The relative quantification (RQ) results were presented as ratios of normalized target gene transcription between the Ab isolates and the Type Strain ATCC 19606 (calibrator), which were obtained according to the following equation: RQ = 2-ΔΔCT, where CT is the value corresponding to the crossing point of the amplification curve with the threshold; ΔCT = target CT or calibrator CT–endogenous CT; and ΔΔCT = target ΔCT—calibrator ΔCT. Reduced *carO* differential transcription of strains relative to that of ATCC 19606 was considered significant when the ratios obtained between RQ values (RQ value of calibrator/RQ value of strains) were ≥2.0 [28], and the overexpression of *adeB*, *adeG* and *adeJ* was considered significant when the ratios obtained between RQ values were ≥4.0 [2]. Each experiment was performed in triplicate in two independent assays.

### Multilocus Sequence Typing (MLST)

The same 10 strains selected for the qPCR reaction were selected for genotyping by MultiLocus Sequence Typing (MLST) as described [29]. The methodology was carried out following the guidelines of the website <https://pubmlst.org/abaumannii/info/primers_Oxford.shtml>. The sequence of amplified internal fragments of housekeeping genes *gltA*, *gyrB*, *gdhB*, *recA*, *cpn60*, *gpi* and *rpoD* was determined and compared with those in the Ab MLST database of Oxford scheme [29]. Clonal complexes (CCs) were formed by Sequence Types (STs) with five or more identical alleles by goeBURST (goeburst.phyloviz.net).

### Whole-genome sequencing

Two strains, one clinical and other environmental, representing prevalent clones previously evaluated by PFGE, were selected for whole-genome sequencing. The total genomic DNA of selected isolates was sequenced, using Illumina NexSeq 500 sequencer (Illumina, San Diego, CA), and the sequence reads were de novo assembled using Velvet pipeline. The pairwise alignment was performed by BLASTn homology searches (https://blast.ncbi.nlm.nih.gov), and in silico comparative analysis using the Center for Genomic Epidemiology (CGE) pipelines. The amino acid sequences of the CarO were aligned using ClustalW with sequences available from GenBank.

### Statistical analysis

Statistical analyses were performed using GraphPad Prism v.5 (GraphPad Software, San Diego, CA). Quantitative assays were compared using one-way analysis of variance (ANOVA) and Bonferroni multiple comparison test. All tests were performed with a confidence level of 95% and statistical significance was defined as P≤0.05.

### Ethical considerations

The data and the samples analyzed in the present study were obtained in accordance with the norms and approved by the Federal University of Uberlandia Ethics Committee (UFU), through license number 228/11.

## Results

The majority (18/23; 78.3%) of strains included in this study were XDR. The presence of associations between IS*Aba1*/OXA-51 and IS*Aba1*/OXA-23 was observed in 21 (91.3%) and 12 (52.2%) strains, respectively. All analyzed environmental strains displayed the IS*Aba1* insertion element linked to both the *bla*_OXA-51_ and the *bla*_OXA-23_ genes (Table 1).

The presence of outer membrane proteins (OMP) genes *carO* and *omp33*-*36*, were detected at 82.6% (19/23) and 100%, frequencies, respectively. The presence of genes encoding the AdeABC (*adeB*), AdeFGH (*adeG*), and AdeIJK (*adeJ*) efflux pumps was evidenced in all analyzed strains. Most exhibited very high MICs for both carbapenems and tigecycline when evaluated by the Etest^®^ method. For the latter antimicrobial drug, the results were confirmed by broth microdilution, and all strains were classified as resistant (Table 1).

**Fig 1:**
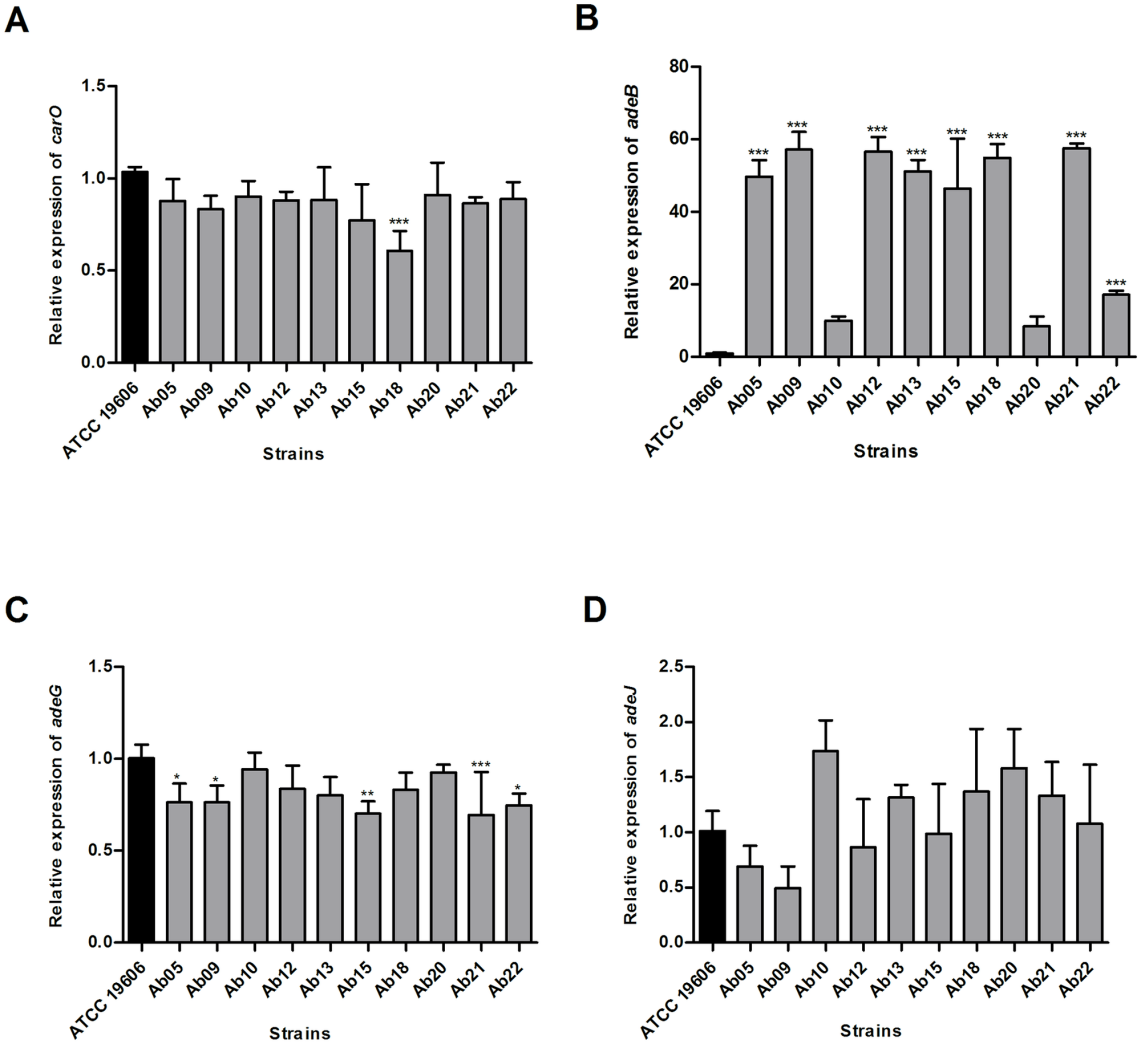
Relative gene expression of the *carO* gene and the genes related to the three efflux pumps (AdeABC, AdeFGH and AdeIJK), determined by qPCR. The results are shown in relation to strain ATCC 19606 used as reference. Each sample was tested in triplicate in two independent assays. Results represent means plus standard deviation (error bars). *P<0.01; ** *P*<0.001; ***P<0.0001, using one-way ANOVA and Bonferroni multiple comparison test.

The expression of the *adeB*, *adeG*, and *adeJ* genes, which encode RND type efflux systems components, and the gene encoding the OMP of 29 kDa (*carO*), were measured by qPCR. The relative gene expression of the tested strains compared to the reference is displayed Fig 1. Based on the transcription levels determined as cut-offs for the hyperexpression of the efflux systems and decreased OMP expression, only the *adeB* gene, which codifies the production of the AdeABC efflux pump, was considered as hyper-expressed. Significant differences in the expression levels of most of the strains in relation to the *adeB* gene were observed (Fig 1B), while the opposite was detected when analyzing the *carO*, *adeG*, and *adeJ* genes (Fig 1A, 1C and 1D).

**Fig 2:**
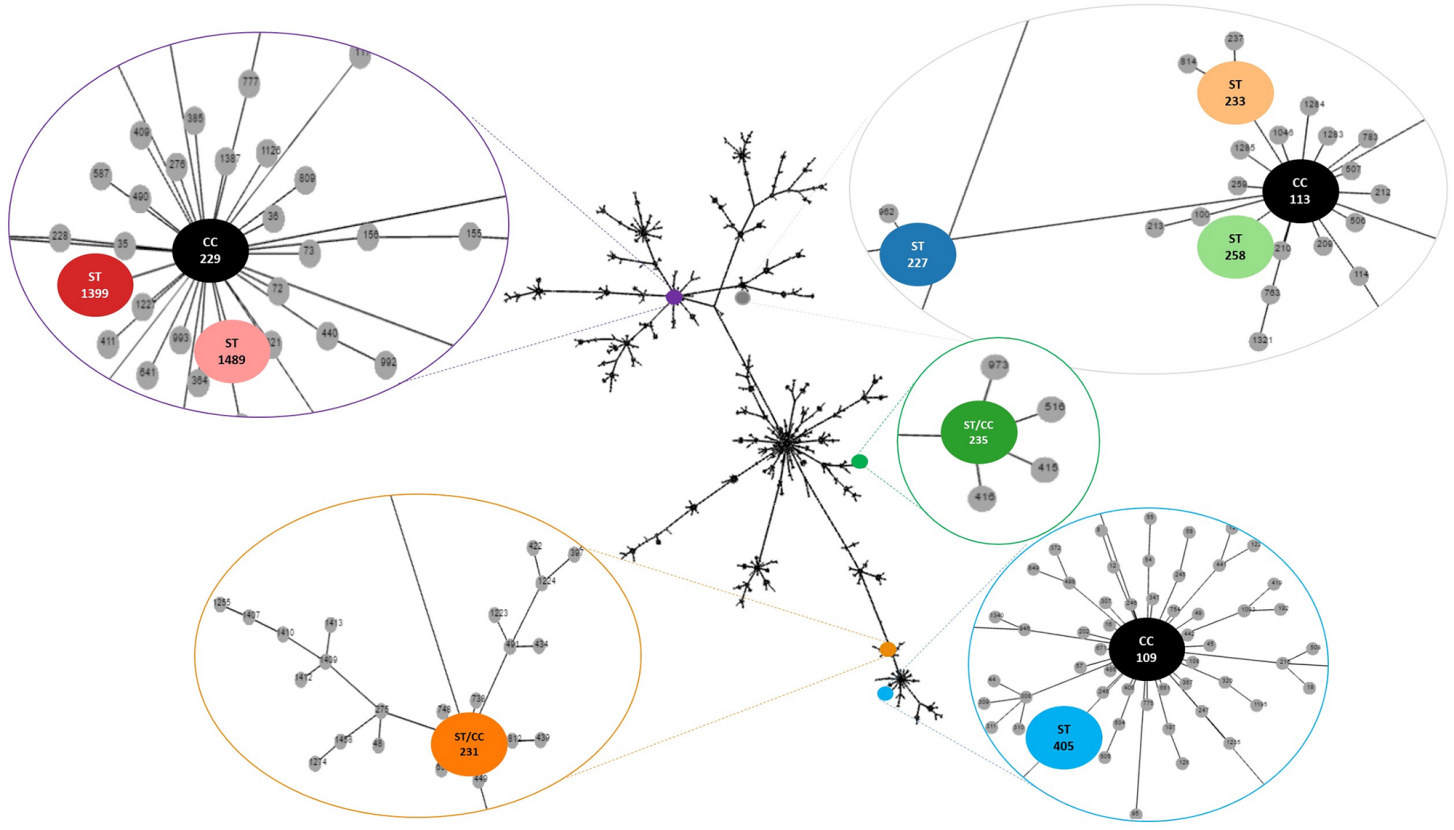
Diagram constructed using the goeBURST algorithm and displayed on phyloviz software (PHYLOVIZ Online) indicating the similarity among sequence types (STs). The Clonal Complexes (CCs) and STs observed in the present study are enlarged and highlighted by color.

MLST revealed eight STs and five CCs (Fig 2), distributed as follows: 1) CC113, ST227 (n = 2), ST233 (n = 1), and ST258 (n = 1); 2) CC229, ST1399, and ST1489; 3) CC109 and ST405 (n = 2); 4) ST/CC235; and 5) ST/CC231. ST1399 is described for the first time herein and was deposited in the MLST database (https://pubmlst.org/bigsdb?db=pubmlst_abaumannii_oxford_seqdef). The three environmental strains included in this analysis belonged to distinct CCs: CC229, CC113, and CC109. CC109 and CC113 were the only CCs presenting strains with the same ST in both environmental and clinical specimens.

**Fig 3:**
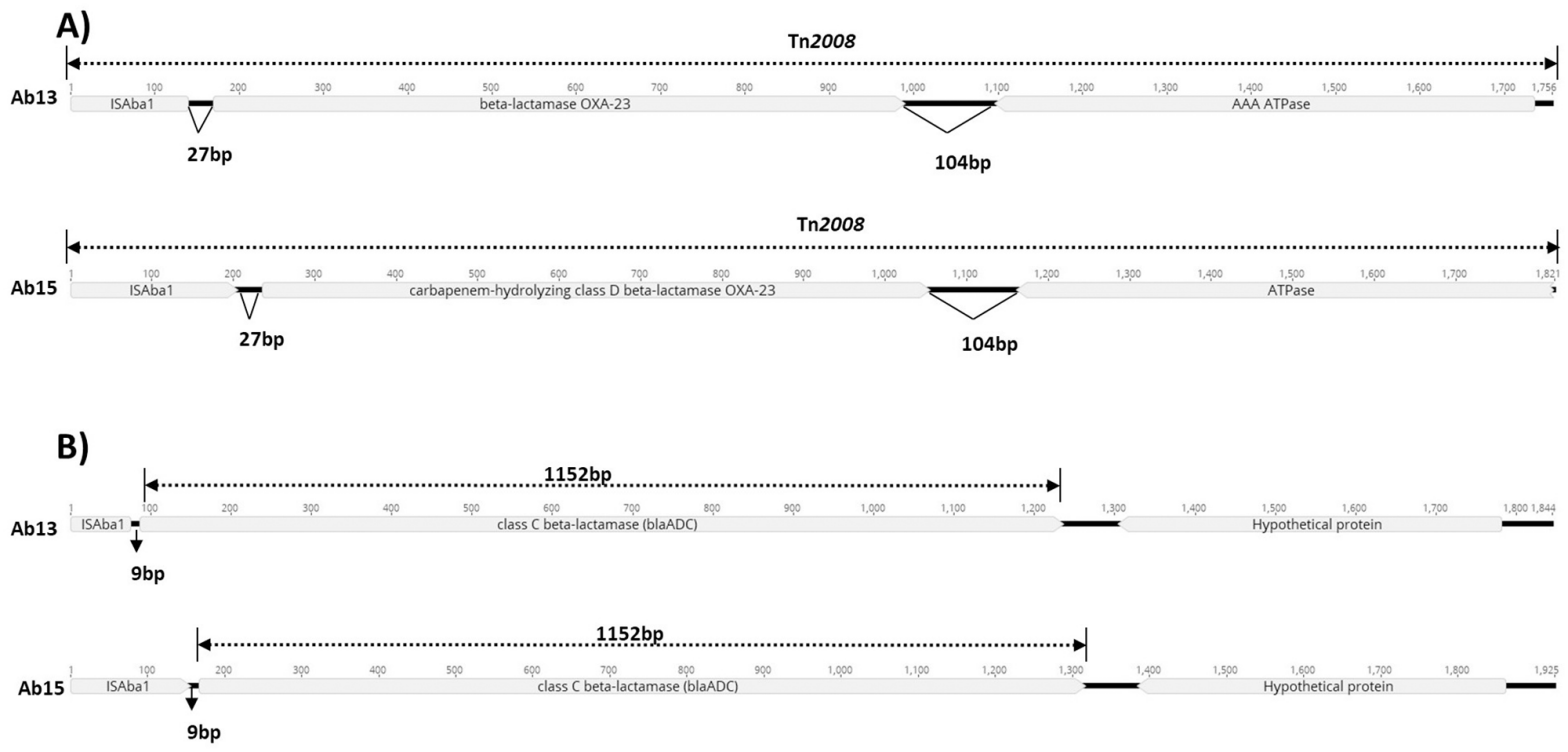
(A) Schematic representation of Tn*2008* with IS*Aba1* located upstream of the *bla*_OXA-23_ gene in the analyzed strains. (B) Schematic representation of the IS*Aba1* localization upstream to the *bla*_ADC-25_ gene.

The total genome belonging to the Ab13 and Ab15 strains exhibited sizes of 3.76 Mb and 3.69 Mb, respectively, and generated a total of 7,694,708 and 9,944,440 reads, respectively, yielding approximately 290X and 370X sequence coverages. The whole genome sequencing analysis confirmed that the Ab13 strain (tracheal aspirate, clone A) belongs to ST/CC231, while the Ab15 strain (environmental, clone H) belongs to ST405/CC109. Resistance to carbapenems was explained by the presence of the Tn*2008* (IS*Aba1*-*bla*_OXA-23_) transposon, located in plasmids (Fig 3A). Both strains exhibited the *bla*_OXA-69_ gene, a variant of the intrinsically encoded *bla*_OXA-51-like_ gene, while third-generation cephalosporin resistance occurs by increased transcription of the *ampC* gene (*bla*_ADC-25_) when associated with IS*Aba1* (Fig 3B).

In the Ab13 strain, resistance to aminoglycosides is justified by the presence of an enzyme that modifies this drug (*aacA4*). In the Ab15 strain, the genes associated to aminoglycoside resistance are *aacA4*, *strA*, and *strB*, and *floR* and *sul2* for chloramphenicol and sulfonamides, respectively. The plasmid-mediated quinolone resistance gene (PMQR) *aac(6')Ib-cr*, was identified in both strains.

**Fig 4:**
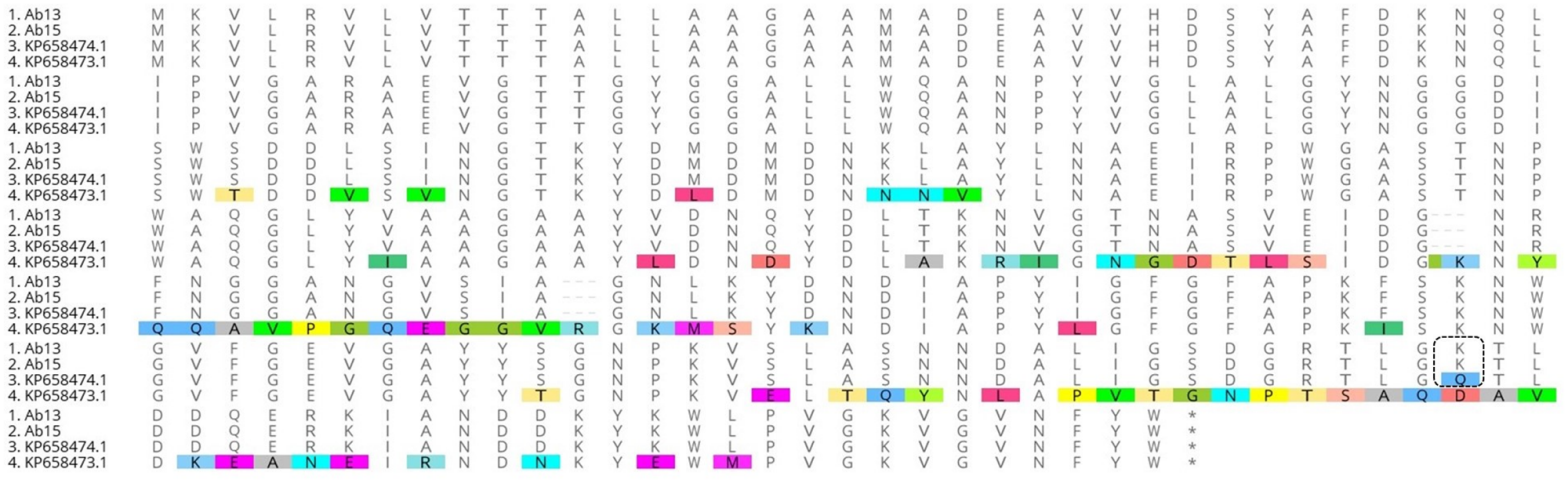
Sequence alignment of *carO* gene for two representative *Acinetobacter baumannii* strains from this study (Ab13 and Ab15) with SJ22 (GenBank: KP658474.1) and ATCC 19606 (GenBank: KP658473.1) strains, indicating homology and difference in amino acids. The alignment was performed using ClustalW. The dotted line indicates the point mutation region (Q218K).

Sequencing of the *carO* gene revealed that both analyzed strains exhibit an isoform protein different from those already registered in the NCBI database (CarOa and CarOb) but with a similarity of 99% to the SJ22 strain registered by Sen and Joshi [30] (Accession number in GenBank KP658474.1), differing only in a change at position 218 (substitution of glutamine for lysine, Q218K) (Fig 4). The strains displayed 93% identity when compared to the reference ATCC 19606 strain (CarOa; accession number in GenBank KP658473), which is susceptible to carbapenems due to the presence of point mutations. In addition, no genetic disruption by IS*Aba1* insertion was observed; therefore, no protein inactivation occurred.

## Discussion

Resistance to antibiotics in Ab has reached alarming levels worldwide, particularly for carbapenems, and strains have been shown to be susceptible only to polymyxins [31–35]. In Brazil, unfortunately, resistance rates to carbapenems are very high (80.7%) [36] and, according to a study carried out by Rossi et al. (2017)[37], a variation of 30 to 70% in resistance to carbapenems was detected in *Acinetobacter* species between 2010 and 2014.

The increase in resistance to carbapenems in clinical Ab strains is mainly associated to the dissemination of OXA-23-producing strains [38–43,26,44], which can be explained by the fact that this gene can also be allocated in plasmids, partly justifying its global reach [45–48]. Allied to this, the presence of specific ISs, such as IS*Aba1*, located adjacent to the *bla*_OXA_ genes, leads to an increase of their expression, resulting in a further decrease in susceptibility to carbapenems [49,4,50–52].

In the present study, approximately half of the analyzed strains were associated to IS*Aba1*/OXA-23, related to high MICs for carbapenems. Viana et al. (2016) [53] observed similar data (i.e., an increase in this association from 22 to 73% between 2009 and 2013, also correlated to elevated MICs for carbapenems. In some of the evaluated strains, no association with either IS*Aba1*/OXA-51 or IS*Aba1*/OXA-23 was observed, despite high MICs, which can be justified by the coexistence of other resistance mechanisms in these strains. These results suggest that the IS*Aba1*/OXA-23 combination may be one of the most significant resistance mechanisms in Ab associated to high XDR phenotypes frequencies and high MICs for carbapenems.

Carbapenem resistance in Ab [54–55] may also result from modifications in the primary structure or loss of outer membrane proteins (OMPs) (porins), such as the 33–36 kDa and the 29 kDa protein named CarO [9]. In the case of OMP CarO, modifications are mostly a result from the rupture of the gene by several insertion elements [56]. From these results, we can infer that the CarO protein is not responsible for carbapenem resistance in the evaluated strains, since no reduced expression levels of this protein were observed. However, the sequence analysis of the *carO* gene revealed a protein isoform different from those already registered in the NCBI database (CarOa and CarOb). A point mutation in the protein (Q218K) has not been reported so far, so it was not possible to determine its exact participation in imipenem resistance.

Another antimicrobial resistance mechanism present in this microorganism is the hyper-expression of efflux pumps, such as those belonging to the RND family (AdeABC, AdeFGH, and AdeIJK) [8]. In addition, AdeABC and AdeFGH play an important role in acquired resistance [57,2], while AdeIJK contributes to intrinsic resistance [58]. Only the *adeB* gene was hyper-expressed based on the qPCR results. According to the literature [7], strains containing the AdeABC pump confer resistance to various antibiotics, including most β-lactams, aminoglycosides, fluoroquinolones, tetracyclines, tigecycline, macrolides, lincosamides, and chloramphenicol. Thus, alongside the presence of IS*Aba1*/OXA-23, this pump is another important mechanism present in the strains analyzed herein.

Tigecycline is an interesting therapeutic option to treat infection by carbapenem-resistant Gram-negative bacteria [59]. However, the isolates evaluated herein demonstrated resistance to this antimicrobial by the Etest^®^ and microdilution methods. A confirmatory evaluation of MICs ≥ 2 μg/ml determined by Etest^®^ is mandatory, as these MICs values could be up to four-fold higher than those obtained by the microdilution method [60,61,62]. The results reported herein corroborate these findings and confirm tigecycline resistance of the strains investigated in this study.

In addition to the genes responsible for carbapenem resistance and high MIC values for tigecycline, most of the strains analyzed by MLST belonged to CC113, confirming its dissemination in Brazil and Latin America [12,63,13–14,64,15–16]. Additionally, a new ST, ST1399, belonging to CC229, is reported. In Brazil, deserves attention another CC identified in carbapenem-resistant Ab strains, CC109, corresponding to the international clone 1 [11,12,64].

Whole genome sequencing of the Ab13 and Ab15 strains demonstrated that carbapenem-resistance is mainly due to the presence of Tn*2008*, which is easily propagated among strains and includes different clones (as evidenced herein) disseminated worldwide [65,66]. In addition, the intrinsic resistance to third generation cephalosporins is due to the increased transcription of the *bla*_ADC-25_ gene, due to the presence of IS*Aba1*, adjacent to the gene, acting as a strong promoter, as described in the literature [67–68,5]. Another detected mechanism was the presence of genes encoding aminoglycoside-modifying enzymes, such as *aacA4* [68,5]. In addition, the PMQR gene *aac(6')Ib-cr* was also identified. This is relevant data, since this gene is well described in Enterobactericeae family members and in *Pseudomonas aeruginosa* [69–71], but has seldom been studied in Ab [72].

## Conclusions

Although a new ST was detected herein in Ab, the present study also observed a wide variety of CCs related to XDR strains carrying the *bla*_OXA-23_ gene, frequently associated with IS*Aba1* (Tn*2008*) as the main carbapenem-resistance mechanism, in addition to the hyperexpression of the AdeABC efflux pump. Understanding resistance mechanisms and the pathogenic potential of this microorganism, both from environmental and clinical origins, aids in explaining its persistence in the hospital environment and can provide tools to improve the treatment of serious infections, as well as increase control and prevention regarding these infections.

## Nucleotide sequence accession numbers

The nucleotide sequence data underlying this study have been uploaded to GenBank under the accession numbers NKKO00000000 (Ab13) and NKKP00000000 (Ab15).

## Supporting information

S1 Table Primer sequences of genes confirmed with PCR and qPCR. (DOCX)
