# Supplementary material for: Molecular characterization and clonal dynamics of nosocomial blaOXA-23 producing XDR Acinetobacter baumannii
Source: PLoS One. 2018 Jun 11;13(6):e0198643. doi: 10.1371/journal.pone.0198643 (PMC5995351; doi:10.1371/journal.pone.0198643)
Supplement: S1 Table — (DOCX) [file pone.0198643.s001.docx]

S1 Table. Primer sequences of genes confirmed with PCR^1^ and qPCR^2^

| **Genes** | **Primers** | **Sequence (5’ – 3’)** | **Reference** |
| --- | --- | --- | --- |
| IS*Aba1*/*bla*_OXA-51_ | ISAba1/OXA-51 F | CACGAATGCAGAAGTTG | [22] |
|  | ISAba1/OXA-51 R | TGGATTGCACTTCATCTTGG |  |
| IS*Aba1*/*bla*_OXA-23-like_ | ISAba1/OXA-23 F | CACGAATGCAGAAGTTG | [4] |
|  | ISAba1/OXA-23 R | ATTTCTGACCGCATTTCCAT |  |
| *omp33-36* | OMP 33-36 F | CATCGATGGCACTAACATGG | [23] |
|  | OMP 33-36 R | AGTGTGACCACCCCAAACAT |  |
| *carO* | CarO F | CATATGAAAGTATTACGTGTTTTAGTG | [24] |
|  | CarO R | GGTACCTTACCAGTAGAAGTTTACACC |  |
| *adeB* | adeB F | TTAACGATAGCGTTGTA | [25] |
|  | adeB R | ACCTGAGCAGACAATGGAATAGT |  |
| *adeG* | adeG F | CTTGCATTTACGTGTGGTGT | [2] |
|  | adeG R | GCTTTTCTACTGCACCCAAA |  |
| *adeJ* | adeJ F | ATTGCACCACCAACCGTAAC | [25] |
|  | adeJ R | TAGCTGGATCAAGCCAGATA |  |
| *adeB* | adeB RT-F | CTTGCATTTACGTGTGGTGT | [2] |
|  | adeB RT-R | GCTTTTCTACTGCACCCAAA |  |
| *adeG* | adeG RT-F | TTCATCTAGCCAAGCAGAAG | [2] |
|  | adeG RT-R | CCTGCTAATGGTAGGGTTAAG |  |
| *adeJ* | adeJ RT-F | GGTCATTAATATCTTTGGC | [2] |
|  | adeJ RT-R | GGTACGAATACCGCTGTCA |  |
| *carO* | carO RT-F | GCAATGGCAGATGAAGC | [26] |
|  | carO RT-R | TAAAGCACCACCGTAACC |  |
| *rpoB* | rpoB RT-F | TCCGCACGTAAAGTAGGAAC | [2] |
|  | rpoB RT-R | ATGCCGCCTGAAAAAGTAAC |  |

^1^PCR, polymerase chain reaction; ^2^qPCR, real time PCR.
